# The molecular puzzle of autoinflammatory skin diseases—a review of chosen conditions

**DOI:** 10.1007/s13353-025-01019-6

**Published:** 2025-10-14

**Authors:** Monika Pieniawska, Karolina Rassek, Tomasz Stein, Adriana Polańska, Aleksandra Dańczak-Pazdrowska, Katarzyna Iżykowska

**Affiliations:** 1 Institute of Human Genetics, Polish Academy of Sciences, Poznań, Poland; 2 Department of Dermatology, Poznan University of Medical Sciences, Poznań, Poland; 3 Department of Dermatology and Venereology, Poznan University of Medical Sciences, Poznań, Poland

**Keywords:** Autoinflammatory skin diseases, Vitiligo, Alopecia areata, Lupus erythematous, Psoriasis

## Abstract

The skin is one of the largest organs in humans and is formed by a layer (termed the epidermis) that enables the body to interact with the environment and protects it from various harmful agents. The epidermis includes a physical, biochemical, and adaptive immune barrier. The immune system in the human body is responsible for protecting organisms from potentially infectious microorganisms, allergens, and toxins, maintaining homeostasis, controlling inflammation processes, and tissue repair. Autoimmune and autoinflammatory diseases are of the immune system manifesting as aberrations in adaptive and innate immunity that lead to an inflammatory state and self-attack damage, also in the skin. The commonness of cutaneous autoinflammations has increased over the past decades, and the occurrence of the disease can have a crucial impact on a patient’s quality of life due to their visible nature, discomfort caused by somatic symptoms, and emotional and social challenges. In this review, we summarize the current knowledge of four common autoinflammatory skin diseases—vitiligo, alopecia areata, systemic lupus erythematosus, and psoriasis—with particular emphasis on their molecular background, including the role of genetic susceptibility, epigenetic regulation, and immunological pathways.

## Introduction

**Fig. 1:**
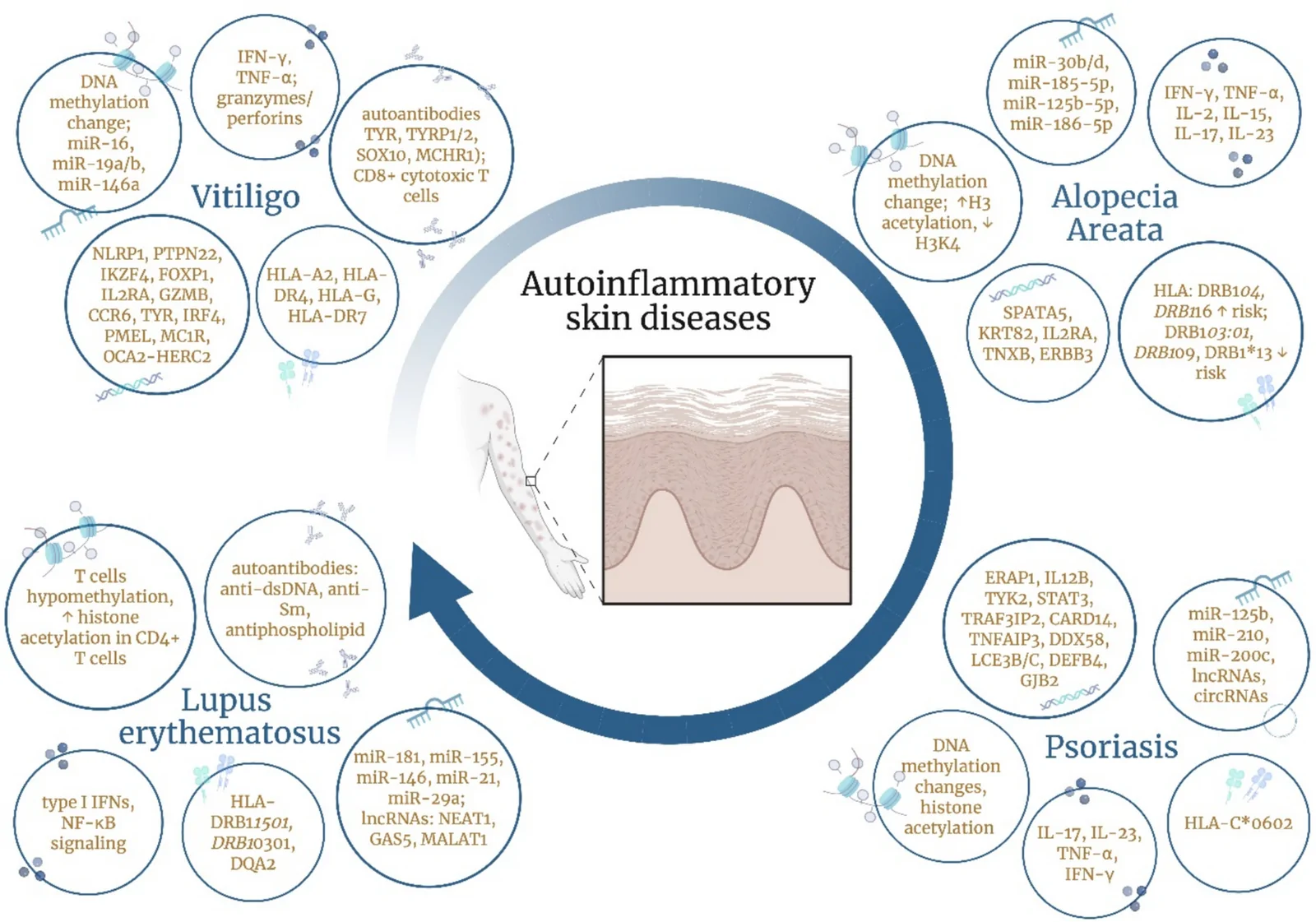
Genetic and autoimmune aspects of autoinflammatory skin diseases. (Created using Biorender.com)

Chronic, autoimmune skin conditions involve immune system dysfunction and inflammation in the skin or other tissues (Howell et al. 2019). In this review, we summarize some of the known genetic and immunologic associations of lupus erythematosus, alopecia areata, psoriasis, and vitiligo, in relevance to the pathogenesis of those diseases. All the above-mentioned conditions involve immune system dysfunction. In each case, the immune system may target specific components of the skin—for example, hair follicles in alopecia areata, keratinocytes in psoriasis, or melanocytes in vitiligo. It may also react to certain triggers in a way that causes skin inflammation. Based on this, we distinguish between organ-specific autoimmune diseases, which are limited to the skin, and systemic autoimmune diseases, such as lupus erythematosus, which can also affect internal organs and, in severe cases, become life-threatening (Fukuyama et al. 2022; Zhou et al. 2022; Marchioro et al. 2022; Li et al. 2022). Immune system involvement contributes to the chronic nature of these diseases, often requiring ongoing treatment to control symptoms and prevent relapses. Furthermore, all of these dermatoses are characterized by the presence of genetic predisposition. Research continues to uncover more about their underlying causes, as advances in one condition’s understanding often contribute to insights into others. Moreover, the multifactorial nature makes these conditions complex to understand and treat, as different individuals might experience varying triggers and responses. In addition, while these conditions share common features, it is also important to recognize their distinct characteristics and unique challenges. Lupus erythematosus, alopecia areata, psoriasis, and vitiligo are relatively rare diseases with incidence rates ranging from 0.1 to 3% (Fig. 1) (Tian et al. 2023; Zhou et al. 2021; Armstrong et al. 2021; Salzes et al. 2016). However, the prevalence of dermatoses has increased over the years in many parts of the world, and the occurrence of the disease can have a profound impact on a person’s quality of life due to their visible nature, the potential for discomfort, and associated emotional and social challenges. Therefore, it seems crucial to understand the exact molecular basis of skin conditions, which might contribute to further understanding and consequently provide safer, more effective, and accurate personalized therapies to manage these conditions.

## Vitiligo

**Fig. 2:**
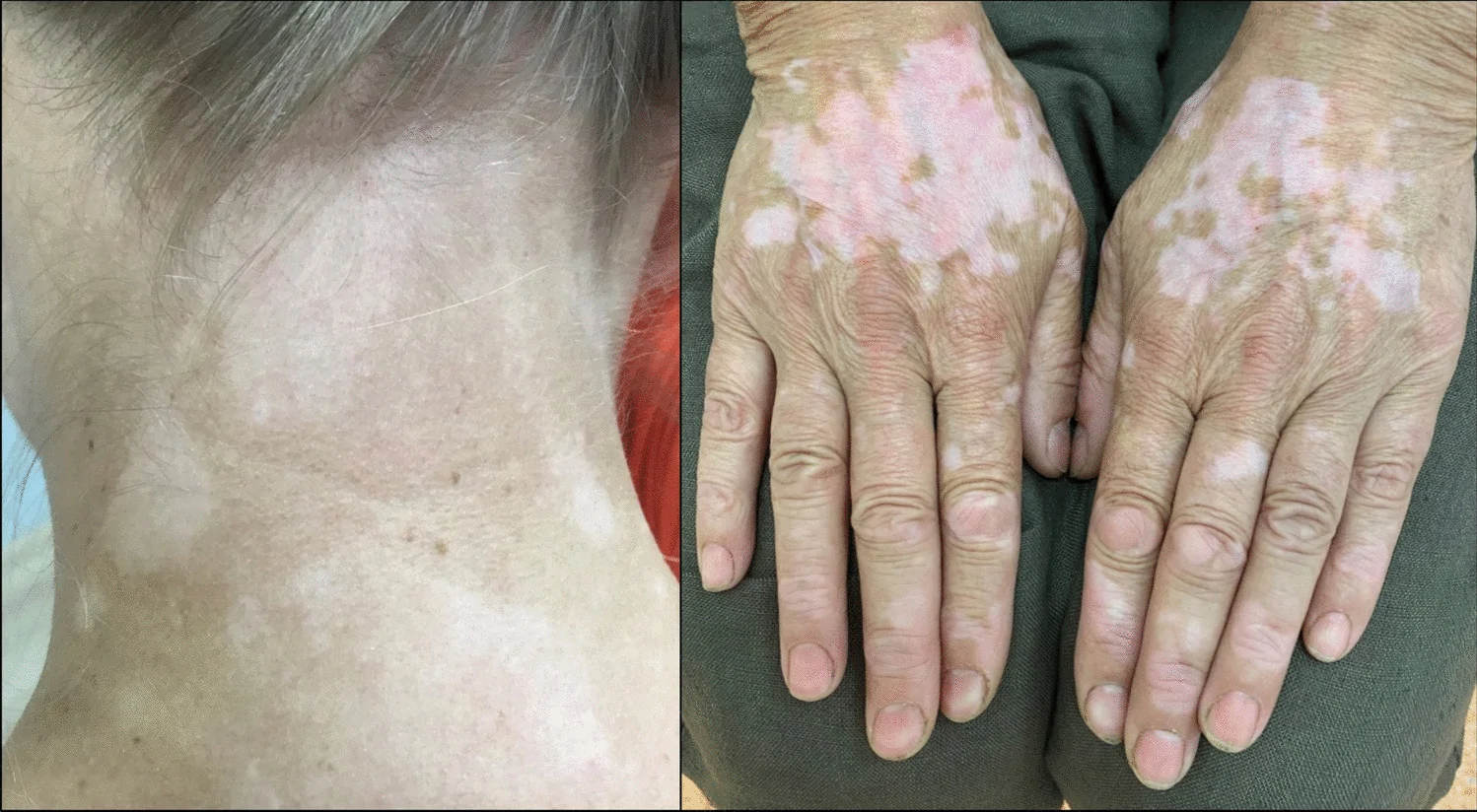
Clinical picture of vitiligo

Vitiligo is an autoimmune skin disease that promotes aggression against melanocytes; therefore, causing depigmentation that appears as white spots (Fig. 2). The disease affects approximately 1% of the world’s population regardless of ethnicity, sex, or geographic region (Salzes et al. 2016). The exact cause of vitiligo is not fully understood, but it is believed to be a multifactorial condition involving genetic, autoimmune, and environmental factors (Fig. 1).

The molecular basis of vitiligo involves such aspects of autoimmune response as the prevalence of autoantibodies; however, their exact role in the development and progression of the disease is still being studied. The hypothesis assumes that autoantibodies against melanocyte antigens that are present in the sera of most vitiligo patients lead to melanocyte destruction through humoral immune mechanisms or cellular immune mechanisms (e.g., cytotoxic T lymphocytes) (Glassman 2011; Farrokhi et al. 2005). The precise role of anti-melanocyte autoantibodies in the pathogenesis of vitiligo has not been established, although both their levels and presence correlate with disease progression and activity (Unal et al. 2021). Since melanin biosynthesis is primarily mediated by the enzymatic activities of tyrosinase (TYR), tyrosinase-related protein-1 (TYRP1), and tyrosinase-related protein-2 (TYRP2), antibodies directed against these melanogenic enzymes have attracted the most attention (Unal et al. 2021; El-Gayyar et al. 2020). Moreover, autoantibodies against transcription factor SOX10 and the melanin-concentrating hormone receptor (MCHR1) have also been demonstrated in the sera of patients with vitiligo (Gottumukkala et al. 2003; Hedstrand et al. 2001). What is more, vitiligo is associated with other autoimmune conditions, especially thyroid diseases, which are characterized by elevated levels of serum antibodies against thyroid peroxidase (TPO) and thyroglobulin (Tg), and those antibodies have also been reported in patients with vitiligo (Vrijman et al. 2012). Studies showed that activation of the native immune system caused by damage to melanocytes affected by oxidative stress promotes cytokine secretion and antigen presentation, which results in activation of the adaptive immune system. Particularly CD8 + cytotoxic T cells are involved in disease pathogenesis, as they are necessary and sufficient for the destruction of melanocytes (Boorn et al. 2009). In the initial active phase of the disease, effector CD8⁺ T cells mediate tissue damage and contribute to the formation of lesions, whereas in the stable phase, resident memory CD8⁺ T cells remain in the affected tissues and play a key role in maintaining the lesions. This mechanism is triggered by the production of inflammatory cytokines like interferon-gamma (IFN-γ) and tumor necrosis factor-alpha (TNF-α) as well as the release of granzymes and perforins, which cause direct damage to melanocytes or induce apoptosis of keratinocytes and melanocytes (Marchioro et al. 2022; Riding and Harris 2019). Studies showed that the production of IFN-γ by CD8 + T cells in the peripheral blood of patients correlates with disease activity, inhibits melanogenesis, or induces apoptosis of the melanocytes (Natarajan et al. 2014; Yang et al. 2015). Moreover, IFN-γ has been shown to induce the chemokine CXCL10, which promotes the migration of autoreactive T cells into the skin and to the epidermis (Rashighi, et al. 2014). TNF-α, a pro-inflammatory cytokine central to many autoimmune diseases, has been associated with the depigmentation process in vitiligo, as it plays a crucial role in melanocyte morphology as well as its function in maintaining IL-1α and IL-6 cytokine balance (Singh et al. 2021).

The risk assigned to the genetics of vitiligo is estimated at 75–83% with several genes associated with the increased susceptibility to the disease, namely *NLRP1*,* PTPN22*, or *IKZF4* (Marchioro et al. 2022). Five genome-wide association studies (GWAS) have been performed for vitiligo in European and Asian populations that identified approximately 50 genetic loci that may increase the risk of disease development. A GWAS study in Caucasians detected associations with at least ten non-MHC loci: *TYR*, *PTPN22*, *RERE*, *FOXP1*, *LPP*, *IL2RA*, *GZMB*, *UBASH3A*, *C1QTNF6*, and *CCR6* (Jin et al. 2010). Parallel GWAS conducted in Chinese populations showed no overlap with susceptibility loci identified in Caucasian cohorts, except for CCR6, the only non-MHC locus that consistently replicated in both groups. This suggests that distinct genetic architectures may underlie vitiligo susceptibility in these populations (Quan et al. 2010). Studies showed that numerous susceptibility genes are shared between vitiligo and other autoimmune diseases, often involving identical high-risk alleles, which is consistent with their observed epidemiological co-occurrence (Quan et al. 2010). One example is the 1858 C > T (R620W) functional polymorphism of *PTPN22*, which encodes a variant of lymphoid tyrosine phosphatase (Lyp) associated with the risk of several autoimmune diseases, including generalized vitiligo. The 1858 T allele has been associated with vitiligo in European populations (English, Romanian, North American, Mexican, and South Indian Tamil cohorts), and genome-wide studies have confirmed its role in Caucasians (Laberge et al. 2008). On the other hand, another meta-analysis did not find a significant association in Asian populations, suggesting population-specific genetic effects (Agarwal and Changotra 2017). Further large-scale studies are needed to clarify the relationship between *PTPN22* 1858 C > T and clinical manifestation of vitiligo, as the immune dysregulation may be caused by the interaction between genetic and environmental factors. Among all identified by GWAS risk alleles were genes regulating adaptive immunity, including *FOXP3*,* CTLA4*,* IL2RA*, or *BACH2*, genes driving innate immunity: *TICAM1*,* IFIH1*, and *CD80*, genes that mediate immune target cell lysis: *GZMB* and *FASLG*, as well as genes directly connected with melanocytes such *as TYR*,* PMEL*,* MC1R*, *OCA2-HERC2*, and *IRF4* (Frisoli et al. 2020). Those melanocyte-specific genes play a key role in the pathogenesis of vitiligo by encoding proteins crucial for melanogenesis and melanocyte survival. Most are preferentially expressed in melanocytes, providing tissue-restricted autoantigens vulnerable to immune-mediated destruction. Polymorphisms in *TYR* influence antigen presentation and immune recognition, while intronic polymorphism in *IRF4* disrupts a melanocyte-specific enhancer bound by *MITF* and *TFAP2A*, reducing *IRF4 *(and downstream *TYR*) expression, therefore further linking pigmentation pathways to immune susceptibility (Jin et al. 2010; Praetorius et al. 2013). Moreover, studies have demonstrated that histocompatibility antigens (HLA) class I and II, like HLA-A2, HLA-DR4, HLA-G, and HLA-DR7, are also correlated with specific subtypes of vitiligo (Singh et al. 2012).

Epigenetic factors also influence the development of vitiligo by affecting gene expression in immune cells and melanocytes. It has been demonstrated that aberrant DNA methylation levels in peripheral blood mononuclear cells (PBMCs) of vitiligo patients control epigenetic regulation of CD8 + T cells and affect the mRNA expression level of *DNMT, MBD, IL-10*, and other genes (Zhao et al. 2010). Recently, using bioinformatic tools, interactions among methylated differentially expressed genes (MDEGs) with different functions and signaling pathways related to the pathogenesis of vitiligo melanocytes have been established. Obtained results uncovered three important functional epigenetic modules, involving a total of 12 (*BCL2L1*,* CDK1*,* ECT2*,* HELLS*,* HSP90AA1*,* KIF23*,* MC1R*,* MLANA*,* PBK*,* PTGS2*,* SOX10*, and* TYRP1*) genes (Pu et al. 2021). Enrichment analysis of these modules showed that MDEGs from module 1 are primarily involved in the cell cycle and cell division, while those in module 2 influenced melanogenesis and pigmentation processes. Module 3 was found to participate in the regulation of cellular stress responses, intrinsic apoptotic signaling pathway, and coordination of complex molecular networks. These findings indicate that MDEGs in vitiligo melanocytes may play regulatory roles in critical biological processes and molecular functions. Nevertheless, several of the genes and pathways unraveled in this study have not yet been investigated as potential therapeutic targets for vitiligo and require further validation.

Multiple microRNAs (miRNAs) have also been demonstrated to act as regulatory factors involved in important aspects of vitiligo pathogenesis, like immune response, growth, differentiation, or apoptosis of melanocytes (Yu et al. 2022). Among the differently regulated miRNAs in PBMCs of vitiligo patients were miR-224−3p, which has been implicated in cytokine production, and miR-3940-5p, involved in stem cell and cancer cell differentiation (Wang et al. 2015). Upregulation of miR-16, miR-19a, miR-19b, miR-146a, and miR-191 was also revealed, and the downregulation of miR-574−3p was correlated with vitiligo lesion severity (Shi et al. 2013) (Table 1).

**Table 1:** Genetic, epigenetic, and immunological features of the discussed autoinflammatory skin diseases

Feature	Autoinflammatory skin disease			
	Vitiligo	Alopecia areata	Lupus erythematosus	Psoriasis
Loss of immune tolerance	Autoantibodies against melanocytes; CD8⁺ T cells targeting melanocytes	CD4 +, CD8 +, NK, mast cells, macrophages, eosinophils infiltration	Autoantibodies (anti-dsDNA, anti-Sm, anti-phospholipids)	Autoreactive cells against keratinocyte antigens
Inflammatory cytokines	IFN-γ, TNF-α	IFN-γ, TNF-α, IL-2, IL-15, IL-17, IL-23	-	IL-17, IL-23, TNF-α, NF-kB signaling
Genetic susceptibility	*NLRP1*, *PTPN22*,* IKZF4*	*KRT82*, *SPATA5*	Polygenic background	12 different *PSORS* loci
HLA association	HLA-A2, HLA-DR4, HLA-DR7	HLA-DRB1*04, HLA-DRB1*16	HLA- DRB1*1501, HLA- DRB1*0301	HLA-C*0602
Oxidative stress	Triggers melanocyte damage and immune activation	Implicated in HF damage and immune activation	-	-
Epigenetic regulation	DNA methylation changes, miR-224-3p, miR-3940-5p, miR-16, miR-19a, miR-19b, miR-146a, and miR-19, miR-574-3p	Increase in DNA methylation and H3 acetylation; decrease in H3K4 methylation, miR-1237, miR-30b/d, miR-548 h-2	Increase in H3, H4 acetylation, DNA methylation changes, miR-181, miR-93, miR-155, miR-146, miR-210, miR-21, miR-29a, miR-15303p, miR-125b, miR-199-3p, lncRNAs (e.g., MALAT1, NEAT1)	DNA methylation changes, histone modifications, miR-125b, miR-210, mir-200c, lncRNAs (e.g., MSX2P1, MIR31HG), circRNAs

## Alopecia areata

**Fig. 3:**
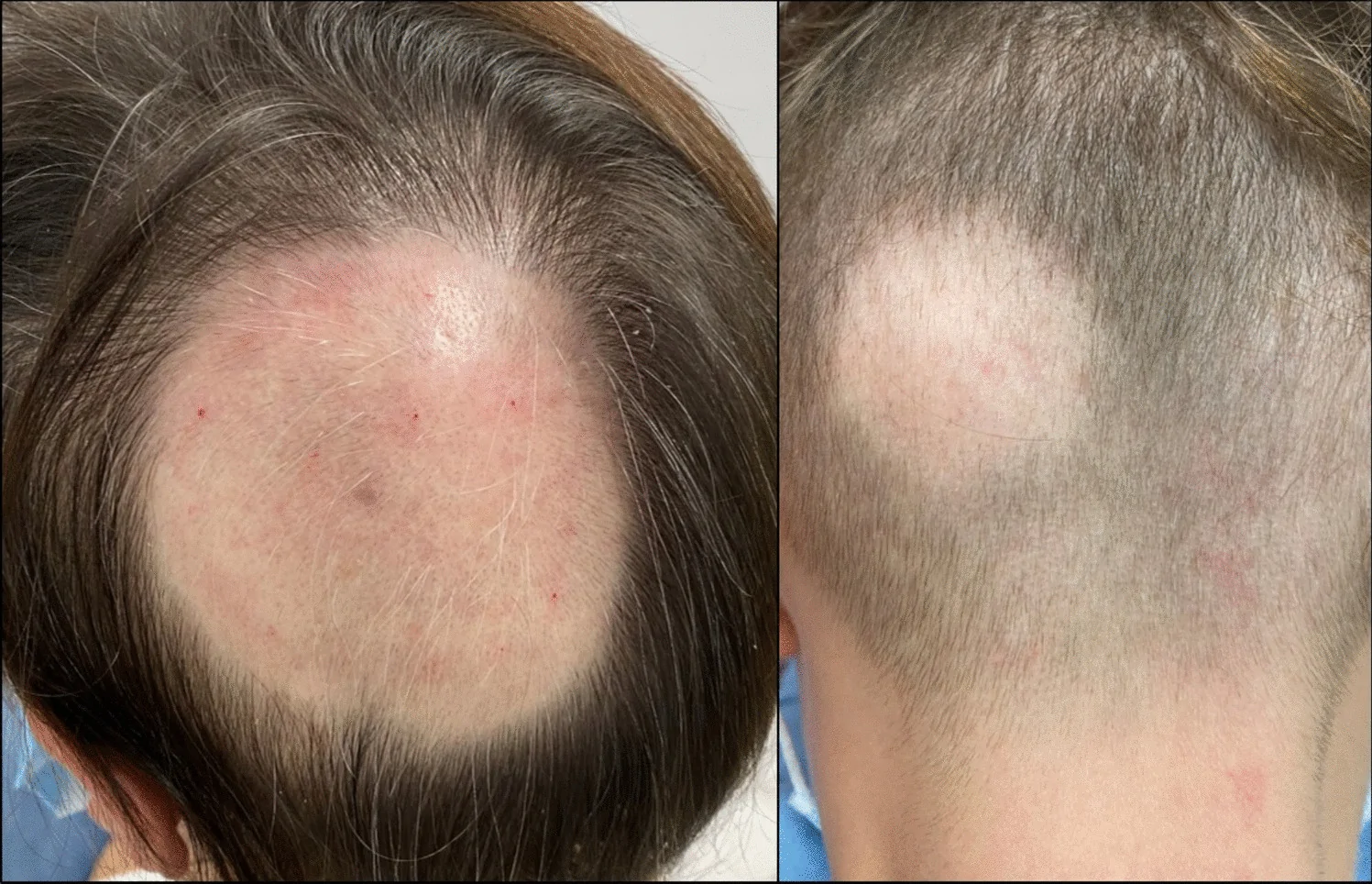
Clinical picture of alopecia areata

Alopecia areata (AA) is a heterogeneous, complex autoimmune skin disease that manifests itself as patches of hair loss on the head and scalp with a variable clinical course (Sutic Udovic, et al. 2024) (Fig. 3). AA in clinical presentations mainly appears as smooth, round, or oval patches of hair loss without atrophy and can be distinguished into other subtypes with a more severe clinical course, such as alopecia totalis (AT), which affects the entire scalp, or alopecia universalis (AU) which involves the entire body surface area. The incidence rate of this condition accounts for 2% of the global population, and the initial diagnosis is usually made based on clinical symptoms and trichoscopic examination, which shows characteristic features such as yellow dots, black dots, or exclamation hairs. The diagnosis is confirmed by histopathological examination (Darwin et al. 2018). The characteristic histological feature of AA is the mononuclear cell infiltration around the hair bulb, which occurs mainly in the acute stage. The infiltrate was described to be made up of CD4 and CD8 T lymphocytes, natural killer cells (NK cells), mast cells, macrophages, and eosinophils (Jabbari et al. 2016; Dy and Whiting 2011). The dystrophic mechanism of hair loss in alopecia areata is the result of rapid anagen arrest, which terminates hair growth (Rakowska et al. 2023).

AA is linked to various gene aberrations correlated with hereditary familial occurrence (Jabbari et al. 2013). Positive family history with clustering, familial aggregation of cases (mainly frequent between siblings), concordance in twins, and HLA studies proved the polygenic molecular background of the disease (Jabbari et al. 2013). The exact etiology of AA is not fully understood, but the immune aggression towards hair follicles (HFs) is caused by genetics, infectious, and environmental factors (Table 1) (Simakou et al. 2019). The autoimmunity of AA seems to be a starting point for insight into the molecular pathogenesis of the condition, so the correlation between AA and HLA seems to be a crucial player in the disease development, which should be emphasized. It was well summarized by Ho CY et al. that HLA-DRB1*04 and HLA-DRB1*16 polymorphisms were associated with increased AA risk, while the risk reduction was associated with HLA-DRB1*0301, HLA-DRB1*09, and HLA-DRB1*13 polymorphisms (Ho et al. 2023a). The role of oxidative stress in AA has also been investigated. Available studies suggest an association between AA and elevated markers, for example, ischemia-modified albumin (IMA), lipid peroxidation products such as malondialdehyde/thiobarbituric acid-reactive substances (MDA/TBARS), and oxidative DNA damage 8-hydroxy-2′-deoxyguanosine (8-OHdG), although the underlying mechanisms remain very complex (Ma et al. 2023; Incel-Uysal et al. 2019; Tanacan et al. 2023; Akar et al. 2002; Cwynar et al. 2018; Mustafa et al. 2021).

In the genetics aspect, the risk of AA occurrence in family-dependent groups, the literature described a notably high incidence rate of AA among first-degree relatives and reported a range between 10 and 42% in observational studies (Biran et al. 2015). Focusing on the genetics of the condition, one of the first GWAS between selected 61 single-nucleotide polymorphisms (SNPs) identified the *SPATA5* (spermatogenesis-associated protein 5) gene locus, which suggests being the susceptibility locus for AA (Forstbauer et al. 2012). Another genome-wide screening on AA indicated 139 genotypes correlated with the disease and 175 SNPs, which were clustered in eight regions correlated with hair follicle-specific genes implemented to AA (Ho et al. 2023b; Petukhova and Christiano 2013). Identification of enrichment pathways based on described GWAS results indicated that genes correlated with AA are associated with the JAK-STAT signaling pathway and other immune-targeting pathways, which correlate clearly with the disease outcome (Petukhova and Christiano 2016). The study based on whole exome sequencing of AA patients revealed one crucial gene, *KRT82*, as a disease-specific one, including its hotspot R47X mutation. *KRT82* expression was decreased in AA patients’ samples and mainly expressed in the anagen phase, proving its biological relevance—reminding that the auto-aggressive attacks of immune cells in AA are described to be present in exactly this phase of hair follicle growth (Erjavec et al. 2022).

Another approach to AA pathogenesis focuses on the impact of stress-induced mechanisms, mainly emphasizing apoptotic and autoimmune pathways. The autoimmune pathway involves the ectopic expression of major histocompatibility complex (MHC) class I in the hair follicle following neurogenic inflammation initiated by various signals, resulting in the decreasing of immune privilege (IP) and forcing CD8 + T lymphocytes to attach to HF keratocytes, while the apoptotic pathway is known to regulate different messengers’ compounds that can promote apoptosis in HF keratocytes (Ahn et al. 2023; Guo et al. 2015).

Besides the genetic alterations in AA, environmental factors and epigenetic modifications are believed to be also responsible for auto-aggressive immune responses in AA (Zhao et al. 2012). Epigenetics has been a focus of studies regarding AA pathogenesis, starting with the analysis of DNA methylation and histone modification patterns and their alterations in patients (Zhao et al. 2012). The comparison of global DNA methylation levels, the methylation levels of histone H3 lysine 4 and lysine 9 (H3K4 and H3K9), and histone H3 and H4 acetylation levels between patients with AA and healthy controls revealed that DNA methylation and H3 acetylation in PBMCs of patients with AA were significantly higher compared to controls, whereas H3K4 methylation was significantly lower (Zhao et al. 2012). Additionally, in the same study, measurement of the expression of *DNMT1*, *MBD1*, *MBD4*, p300 (*EP300*), histone deacetylase 1 (*HDAC1*), SET7/9 (*SETD7*), G9A (*EHMT2*), JMJD2C (*KDM4C*), and JARID1A (*KDM5A*) genes was indicated to increase, while the expression of *HDAC2*, *HDAC7*, LSD1 (*KDM1A*), JMJD2A (*KDM4A*), and JMJD2B (*KDM4B*) genes decrease in AA patients (Zhao et al. 2012). Moreover, the role of inflammatory cytokines is known to be engaged in the development of the disease. The key cytokines include IFN-γ, TNF-α, interleukin-2 (IL-2), interleukin-15 (IL-15), interleukin-17 (IL-17), and interleukin-23 (IL-23) (Agamia et al. 2020; Ebrahim et al. 2019; Kasumagić-Halilovic et al. 2018; Tomaszewska et al. 2022; Ortolan et al. 2019).

Moreover, miRNAs are known to play a key role in other autoinflammatory skin diseases such as psoriasis or atopic dermatitis, and their role in AA has also been investigated. It was discovered that three miRNAs—miR-1237, miR-30b/d, and miR-548 h-2—were associated with AA (Ho et al. 2023b; Tafazzoli et al. 2018; Sheng et al. 2019). miR-30b/d showed lower expression in the hair follicle of AA patients and was described to be the most significant one for the later analyses. Target gene analyses for these three miRNAs indicated up to 42 significantly associated target genes, including *IL2RA*, *TNXB*, and *ERBB3*, which had been identified as susceptibility loci in previous genome-wide association studies (Tafazzoli et al. 2018). Another study on blood-dysregulated miRNAs indicated that miR-185-5p, miR-125b-5p, and miR-186-5p are good candidates to play synergistic roles in the active phase of AA, which could lead to the discovery of more promising treatment in the future (Sheng et al. 2019). These findings provide novel insights into the pathogenesis of AA and set a solid background for understanding the influence of epigenetic factors in the development of AA, which could lead to new therapeutical strategies in the future.

## Lupus erythematosus

**Fig. 4:**
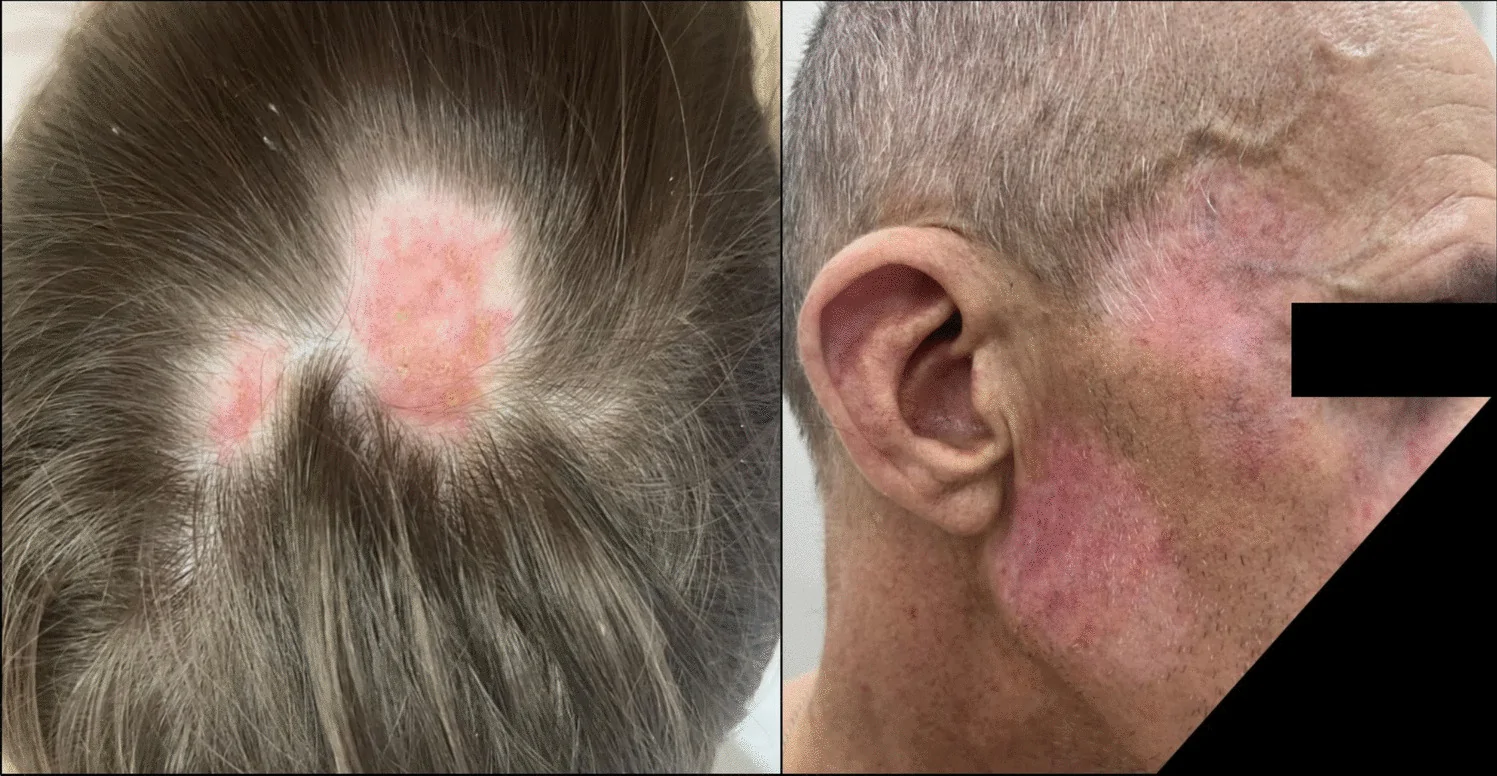
Clinical picture of discoid lupus erythematosus

Systemic lupus erythematosus (SLE) is an autoimmune disease characterized by the presence of antibodies directed against the body’s own antigens, particularly those located in the cell nucleus (Woźniacka et al. 2018) (Fig. 4). SLE can manifest a wide spectrum of symptoms, from skin lesions to systemic organ disease, involving major internal organs. (Wenzel 2019). The disease can be distinguished into various subtypes, including acute cutaneous lupus erythematosus, subacute cutaneous lupus erythematosus, and chronic cutaneous lupus erythematosus (Żychowska and Reich 2022). Chronic cutaneous lupus erythematosus includes discoid lupus erythematosus (DLE), lupus erythematosus profundus (LEP), chilblain cutaneous lupus (CHLE), and lupus tumidus (LET) (Okon and Werth 2013). The diagnosis of SLE is based on the clinical examination and presence in blood serum SLE-specific antibodies, which include antibodies directed against native DNA (anti-double-stranded DNA—anti-dsDNA), antibodies reactive with the Sm antigen (anti-Sm), or antiphospholipid antibodies (Woźniacka, et al. 2018). SLE is a very complex and clinically challenging condition, and its pathogenesis is not fully known.

The molecular background of the disease seems to be multifactorial and focuses mainly on the influence of immunologic self-tolerance breakdown and genetics (Ghodke-Puranik and Niewold 2015). The risk is attributed to genetic factors, immune disorders, environmental factors, and their interaction (Table 1). The most important environmental factor causing the pathological process is UV radiation, both in the UVB and UVA range (Woźniacka, et al. 2018). Many studies refer to the high impact of HLA complexes on SLE pathogenesis. Studies on the importance of autoinflammatory conditions in HLA regions have shown that genes in class II are represented as SLE susceptibility loci (Ghodke-Puranik and Niewold 2015). Previous studies of the HLA region in systemic lupus erythematosus have shown that HLA-DRB1 (DRB1*1501 and DRB1*0301) is strongly associated with the condition (Ghodke-Puranik and Niewold 2015; Connolly and Hakonarson 2012). A study performed by Chung SA. et al. investigating anti-dsDNA negative SLE cases in comparison to healthy controls identified a significant association with the SNP rs2301271, located 9 kb downstream of HLA-DQA2 (Chung et al. 2011). Additionally, a strong correlation was observed between anti-dsDNA and HLA-DR3 at rs2187668, reinforcing the critical role of the HLA region in shaping autoantibody responses. Consistently, the largest recent genetic association study on SLE sub-phenotypes further demonstrated that HLA-DRB1*03:01 not only contributes to SLE susceptibility but may also have a broader influence on disease pathogenesis (Morris et al. 2014). Compared to the important role of HLA in the pathogenesis, the familial aggregation screening provided that siblings of SLE patients have a greater risk of developing the condition, with almost 66% heritability in conducted twin studies so far, emphasizing the non-HLA contribution in the case of susceptibility (Kwon et al. 2019; Lawrence et al. 1987). The polygenic background of systemic lupus erythematosus is well known, as evidenced by the large number of genetic loci associated with this disease. It has been summarized by Kwon YC et al. that by this time, more than a hundred non-HLA loci have been identified as SLA-susceptibility associated (Kwon et al. 2019).

Besides identifications of many systemic lupus erythematosus-involved genes, which can be considered disease predisposing, epigenetic factors seem to be important in case of susceptibility to developing the condition. Studies focused on DNA methylation in monozygotic twins discordant for SLE have indicated significant differences in these discordant pairs (Javierre et al. 2010). Other differences in epigenetic modifications, such as DNA methylation, histone modifications [acetylation, ubiquitination, phosphorylation, and citrullination of histone tails], miRNAs, and non-coding RNAs, can alter the expression and function of genes involved in SLE pathogenesis (Ghodke-Puranik and Niewold 2015; Javierre et al. 2010). A study on histone deacetylase inhibitors in mice was found to alleviate the symptoms and overall severity of the disease (Mishra et al. 2003; Ren et al. 2019). Higher histone H3 and H4 acetylation were detected in human SLE CD4 + T cells and negatively correlated with SLE disease activity suggesting a potential role for abnormal histone acetylation in SLE pathogenesis (Mishra et al. 2003; Hu et al. 2008). In the case of DNA methylation, during its inhibition in mice and humans, the induction of T-cell autoreactivity was indicated (Quddus et al. 1993; Yung et al. 1997). Moreover, T cells isolated from systemic lupus erythematosus patients had lower methylation levels than control T cells from healthy individuals, which emphasizes a potential role for altered methylation in the SLE molecular background and also a relationship between DNA methylation and autoimmunity (Richardson et al. 1990). Single loci methylation analysis identified severe differentially methylated genes in systemic lupus erythematosus patients, for example, *PRF1* (demethylation of the promoter),* ITGAL* (hypomethylation of the promoter), and *TNFSF7* (demethylation) (Kaplan et al. 2004; Lu et al. 2002; Lu et al. 2005). Also, studies highlighted miRNAs as potential biomarkers for autoinflammatory diseases, including systemic lupus erythematosus. Afrashteth Nour et al. summarized findings from previous studies and reported up to ten miRNAs significantly associated with SLE, including miR-181, miR-98, miR-155, miR-146, miR-210, miR-21, miR-29a, miR-153-3p, miR-125b, and miR-199-3p (Afrashteh Nour et al. 2023). Additionally, to these findings, 12 long non-coding RNAs (lncRNAs)—NEAT-1, TUG1, GAS5, linc0597, lnc-DC, linc0949, lnc0640, lnc3643, lnc5150, lnc7514, lncagf, and MALAT1—were summarized to be correlated with the disease (Afrashteh Nour et al. 2023). In summary, altered immune tolerance and interaction between SLE susceptibility genes and various epigenetic factors seem to be crucial players, resulting in pathogenic autoantibody production, which is the main factor of systemic lupus erythematosus development.

## Psoriasis

**Fig. 5:**
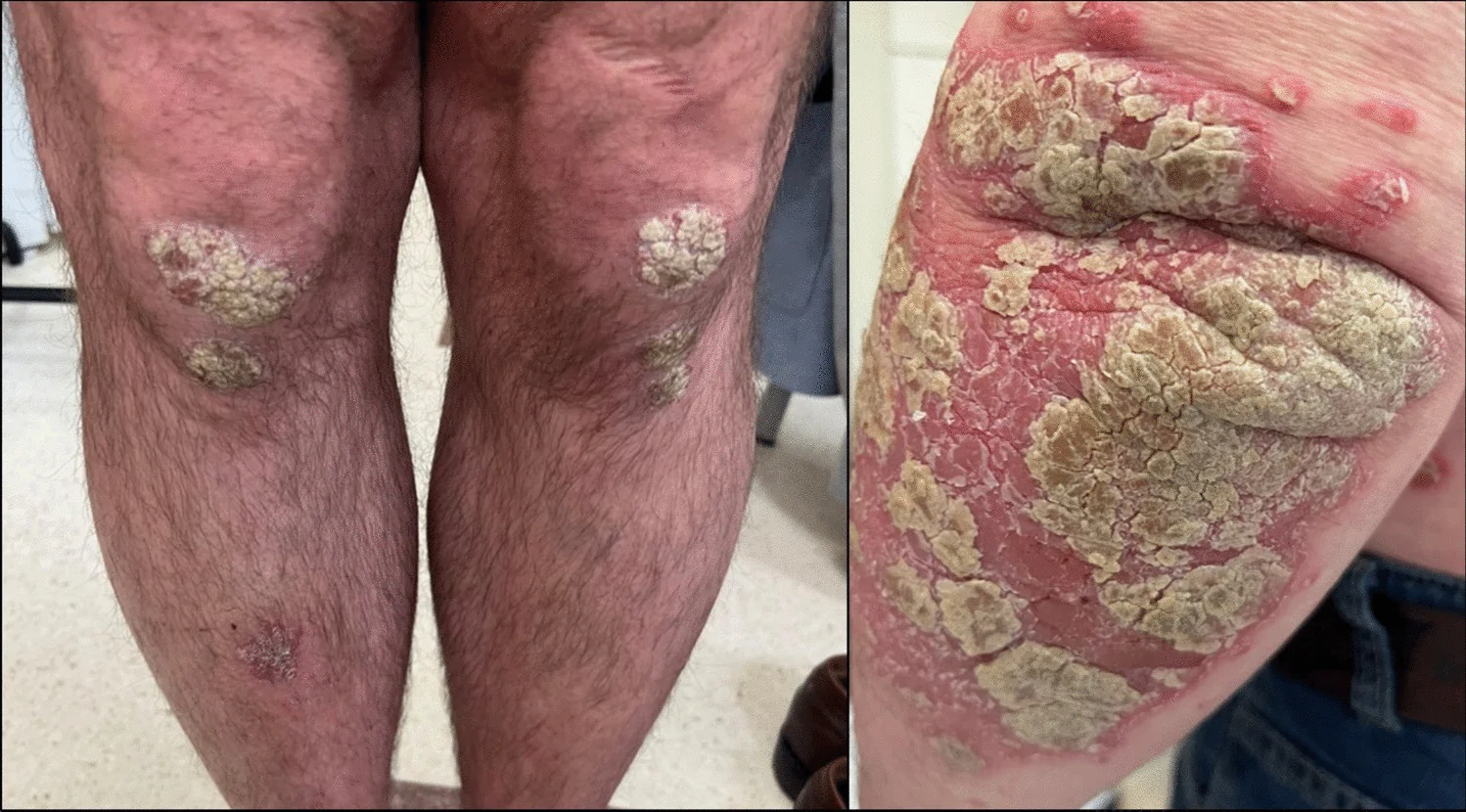
Clinical picture of psoriasis

Psoriasis is a chronic, inflammatory skin disease, affecting about 2–3% of the worldwide population (Harden et al. 2015). The treatment available is only symptomatic while the disease has a great negative impact on the patient’s quality of life (Griffiths et al. 2021). Psoriasis has different clinical phenotypes, but the most frequent and easily recognized is chronic plaque, also called psoriasis vulgaris, characterized by demarcated, erythematous, scaly plaques affecting any skin site, including the scalp (Griffiths et al. 2021) (Fig. 5). From a pathogenic perspective, psoriasis lies at the interface between autoimmune and autoinflammatory disorders, unlike other classical autoimmune disorders described in this paper. The main difference between autoinflammatory and autoimmune disorders is that the former stem from innate immune dysfunction, often triggered by genetic mutations, endogenous danger signals, or cytokine dysregulation, whereas the latter arise from adaptive immune dysfunction involving autoreactive T or B cells (Kim 2023; Liang et al. 2017). In psoriasis, both autoimmune and autoinflammatory mechanisms frequently coexist, with each potentially triggering or amplifying the other.

The actual pathogenesis is still not known, as psoriasis is a complex disorder influenced by not only genetic and epigenetic factors but also environmental factors that can trigger the disease symptoms (Table 1). Genetic factors play a significant role in the development of psoriasis, and having a family history of psoriasis increases the risk of developing the condition (Griffiths et al. 2021). Multiple genes have been identified that contribute to an individual’s susceptibility to the disease, as well as increasing the risk of disease onset. Areas thought to harbor psoriasis-related genes with psoriasis susceptibility were initially named PSORS (psoriasis-susceptibility) loci, and 12 different PSORS loci were mainly identified at first (Harden et al. 2015), which was followed by the identification of more candidate genes based on GWAS (Ray-Jones et al. 2016). These are mainly genes involved in antigen presentation, skin barrier function, and innate and adaptive immune system, including interleukin, interferon, and NF-kB signaling (Mateu-Arrom and Puig 2023). Recently, the role of epigenetic factors, including miRNAs, lncRNAs, methylation, and modification of histones, has been also investigated and emphasized (Mateu-Arrom and Puig 2023).

The best-defined psoriasis triggers include exposure to environmental pathogens, drugs, alcohol consumption, smoking, and stress, while in terms of genetic susceptibility, over 80 different genetic loci that increase the risk of psoriasis onset have been identified so far (Griffiths et al. 2021). Conversely, certain environmental and lifestyle factors may attenuate psoriasis severity. For example, controlled exposure to ultraviolet (UV) light, weight reduction in obese patients, smoking cessation, moderation of alcohol intake, stress management, and a healthy diet have all been reported to reduce disease burden (Barrea, et al. 2016; Ko et al. 2019; Enamandram and Kimball 2013).

With the right trigger in a genetically susceptible individual, a psoriasis plaque develops as a result of the overactive response of dendritic cells, certain T cell subsets, particularly Th1 and Th17 cells, and keratinocytes (Griffiths et al. 2021). The excessive production of pro-inflammatory cytokines like IL-17, IL-23, and TNF-α promotes inflammation and triggers the immune response (Fig. 1). Keratinocytes, activated by immune signals, hyperproliferate and release chemokines and cytokines that attract more immune cells to the skin, including neutrophils that release enzymes and other factors that further contribute to inflammation and lead to tissue damage. As a result, complex, interacting, and self-sustaining inflammatory circuits are created in psoriatic lesions (Griffiths et al. 2021).

The major genetic risk factor for early-onset psoriasis is the HLA-C*0602 allele localized within the major histocompatibility complex (MHC) region, within the so-called *PSORS1* locus (Fig. 1) (Ray-Jones et al. 2016; Nair et al. 2006). It was found in about 20–50% of psoriasis cases, but only in about 4–16% of healthy controls (Harden et al. 2015). The inheritance of this allele increases the risk of psoriasis by four to five times, but interaction with another risk variant of the *ERAP1* gene, which is involved in processing antigens for HLA class I presentation, further increases that risk (Analysis, et al. 2010). These associations suggest that psoriasis could be caused by a T-cell-initiated response to an autoantigen that is exhibited on HLA-C*0602 and processed by a specific ERAP1 variant; however, no specific “auto-antigen” has been confirmed yet (Harden et al. 2015).

Many genes related to immune system functions, such as cytokines and cytokines signaling pathways, are implicated in psoriasis (Ray-Jones et al. 2016; Mateu-Arrom and Puig 2023). These include genes involved in NF-kB signaling (*TNFAIP3*,* TNFRSF1B*,* TRAF3IP2*,* TNIP1*,* NFKBIA*, and *CARD14*) that regulate multiple aspects of innate immunity and interferon and antiviral response pathways (*DDX58*,* IFIH1*) (Nair et al. 2009; Tsoi et al. 2012; Stuart et al. 2010). Moreover, genes involved in Th1 signaling, mostly related to the IL12 and IL2 pathways, including *IL12B*, encoding a p40 subunit of IL12, *TYK2*, which encodes a downstream molecule of IL12 receptor, and *STAT5A/B* and *ILF3* involved in IL2 production (Mateu-Arrom and Puig 2023; Analysis, et al. 2010).

The crucial role in psoriasis is related to IL23 affecting Th17 functioning. Several SNPs associated with psoriasis risk have been found in genomic regions corresponding to IL-23 subunits p19 and p40 and IL23 receptor (*IL23R*) (Analysis, et al. 2010) and its downstream molecules like TYK2, JAK2, and STAT3 (Ellinghaus et al. 2012). Other genes associated with psoriasis are involved in Th17 differentiation like *SOCS1* and *ETS1* and in IL17 production and pathway, including IL17 receptor *IL17RD*, *TRAF3IP2* gene, and *KLF4* (Mateu-Arrom and Puig 2023; Lambert et al. 2019).

Not only altered genes associated with the immune response contribute to psoriasis risk but also those that are involved in skin barrier function and keratinocyte differentiation (Mateu-Arrom and Puig 2023). Polymorphisms in skin barrier regulatory genes such as defensins (*DEFB4*) (Hollox et al. 2008), late cornified envelope (*LCE3B* and *LCE3C*) genes (Li et al. 2011), and connexin (*GJB2*) (Sun et al. 2010) have been identified. The downregulation of filaggrin (FLG) expression in psoriatic skin was also detected, yet loss-of-function variants of the FLG gene were not associated with psoriasis susceptibility (Huffmeier et al. 2007).

Furthermore, numerous epigenetic factors contributing to psoriasis have been identified (Fig. 1) (Mateu-Arrom and Puig 2023) including miRNAs, lncRNAs, and circRNAs. Deregulated non-coding RNAs usually influence keratinocyte proliferation and differentiation, inflammation, and immune response. Among many, the most deregulated miRNAs in psoriatic lesions are miRNA125b (Wei et al. 2017) and miR-210 (Wu et al. 2018), while the elevated expression of another miR200c, both in the plasm and lesional skin from psoriasis patients, was positively correlated with disease severity (Magenta et al. 2019). Among lncRNAs highly upregulated are RP6-65G23.1, MIR31HG, and MSX2P1, while circRNAs are less abundant in psoriatic skin compared to non-lesional and healthy skin, and two of them, ciRS-7 and circZRANB1, were identified as promising psoriasis diagnostic biomarkers (Zeng et al. 2021).

Several whole-genome DNA methylation analyses showed hyper- and hypomethylation of the gene regions in the skin from the psoriatic lesions and in the PBMC from psoriatic patients (Mateu-Arrom and Puig 2023). Altered methylation was detected to affect the expression of several PSORS genes, such as *S100A9*,* PTPN22*,* SELENBP1*,* CARD14*, and *KAZN* (Chandra et al. 2018). Moreover, hypermethylation was detected in the promoter region of the known tumor suppressor gene *p16INK4a*, which was negatively related to disease severity (Chen et al. 2008), while *CYP2S1*, involved in the metabolism of retinoic acid and NF-kB pathway, was one of the most significantly hypomethylated and, as a result, upregulated genes identified in the psoriatic skin samples (Zhou et al. 2016). Other studies showed that changes in the methylation status could have been initiated due to alterations in genes involved in methylation regulation, like *DNMT1*, which is overexpressed in PBMC from psoriatic patients, or methyl-CpG binding domain protein 2 (*MBD2*) and methyl-CpG binding protein 2 (*MeCP2*), both being appropriately downregulated (Zhang et al. 2010). Another epigenetic factor, histone modifications, has also been implicated in psoriasis (Mateu-Arrom and Puig 2023). Histone methylation was identified to be important in regulating cytokine production, including IL23, and drug responses in psoriasis (Zeng et al. 2021), while histone acetylation within the *IL17A* region was proved to induce Th17 differentiation (Xia et al. 2020). Moreover, a histone deacetylase HDAC1, involved in the regulation of many genes, was detected to be upregulated in psoriatic skin (Tovar-Castillo et al. 2007).

It is worth mentioning that the research on understanding the genetic background of psoriasis has provided valuable insights into the disease’s pathogenesis and has led to the development of targeted therapies, such as biological drugs that specifically target immune-related molecules (Harden et al. 2015). However, more genetic research is still needed to fully understand the disease and further improve the treatment.

## Discussion/summary

In summary, this review emphasizes the wide diversity of molecular genetic and epigenetic mechanisms involved in the pathogenesis of four different autoimmune skin diseases encountered in everyday dermatology practice—vitiligo, alopecia areata, lupus erythematosus, and psoriasis. Although these diseases have distinct phenotypes, they share a common cause: immune dysregulation mediated by multiple genes (polygenic), epigenetic changes, and environmental factors (Table 1). Genetic predisposition, supported by family history and genomic research, highlights the hereditary risks, while convergent inflammatory circuits drive tissue-specific autoimmunity against melanocytes, hair follicles, keratinocytes, and other targets. The involvement of the immune system—or rather its dysfunction—seems to be the starting point on the very complicated correlation map between each of the diseases mentioned, and understanding the mechanisms that cause autoimmunity against healthy cells may contribute to better development of disease-specific prevention tools in high-risk groups and beneficial treatment options that can alleviate troublesome symptoms. Although no single susceptibility gene has been identified for vitiligo, alopecia areata, psoriasis, or SLE, genetic studies have demonstrated the important role of both genetic and epigenetic factors. They have also shown that a family history of these diseases is associated with an increased risk of developing all described conditions. Identification of candidate genes could lead to earlier detection, improved risk assessment, and the development of targeted therapies, shedding better light on preventive aspects, for example, personalized lifestyle changes to prevent symptoms. As also described in this review, each disease is believed to be polygenic, which raises the question of how much specific genes interact with each other and thus translate into the severity of symptoms and the aggressiveness of the disease. Identification of specific markers for a given disease would be a key player in diagnosis, prevention, and predictability of an individual’s response to a specific treatment and preventing relapses. From a clinical perspective, the most important question seems to be how to develop more accurate and reliable methods for early diagnosis and prediction of disease progression. Early intervention can significantly improve outcomes and reduce disease recurrence. To fully answer them, ongoing research and collaboration between scientists, clinicians, and patients is necessary. An exact understanding of the interplay of genetic and environmental factors in autoimmune skin diseases, such as vitiligo, alopecia areata, lupus erythematosus, and psoriasis, may lead to the discovery of personalized diagnostic and therapeutic approaches, improving specificity and effectiveness, which would be a huge benefit for patients who struggle every day with troublesome symptoms that worsen their quality of life.

## Data Availability

Not applicable.
